# Establishing a birth cohort to investigate the course and aetiology of asthma and allergies across three generations – rationale, design, and methods of the ACROSSOLAR study

**DOI:** 10.1186/s12889-015-2555-y

**Published:** 2015-12-04

**Authors:** Tobias Weinmann, Jessica Gerlich, Sabine Heinrich, Dennis Nowak, Jennifer Gerdes, Jenny Schlichtiger, Erika von Mutius, Bianca Schaub, Christian Vogelberg, Diana Roller, Katja Radon

**Affiliations:** Occupational and Environmental Epidemiology & NetTeaching Unit, Institute and Outpatient Clinic for Occupational, Social and Environmental Medicine, University Hospital of Munich (LMU), Ziemssenstr. 1, 80336 Munich, Germany; Institute and Outpatient Clinic for Occupational, Social and Environmental Medicine, University Hospital of Munich (LMU), Ziemssenstr. 1, 80336 Munich, Germany; Dr. v. Haunersches Kinderspital, University Hospital of Munich (LMU), Lindwurmstr. 4, 80337 Munich, Germany; Paediatric Department, University Hospital Carl Gustav Carus Dresden, TU Dresden, Fetscherstr. 74, 01307 Dresden, Germany; German Centre for Lung Research (DZL), Munich, Germany

**Keywords:** Atopic disease, Asthma, Allergy, Birth cohort, Epigenetics, Environmental health, DNA methylation, Respiratory tract diseases, Cross-generational effects

## Abstract

**Background:**

Atopic diseases are a major burden of disease on a global scale. Regarding their aetiology, the early years of life are assumed to play a crucial role. In addition, there is growing evidence that elucidating the impact of cross-generational effects and epigenetic mechanisms such as DNA methylation can substantially widen the scientific knowledge of the occurrence and progression of these diseases. We are thus aiming at following the course of asthma, allergies, and potential risk factors for their occurrence across three generations by establishing a birth cohort in the offspring of an existing population-based cohort.

**Methods/Design:**

2051 young adults who have been recruited in 1995 for Phase II of the International Study of Asthma and Allergies in Childhood (ISAAC) and who have subsequently been followed-up by the Study on Occupational Allergy Risks (SOLAR) are asked bi-annually since 2009 if they conceived a child in the meantime. If parenthood is reported, parents are invited to enrol along with their children in the ACROSSOLAR cohort. Participation involves completing a questionnaire assessing general and health-related information about the course of the pregnancy and the first year of life of their children. Subsequently, the children are followed up until primary school age when asthma and allergies can be diagnosed reliably. In addition, DNA for epigenetic analysis will be collected and analysed. Longitudinal data analysis techniques will then be used to assess potential associations between early-life exposures and onset of childhood asthma and allergies taking into account epigenetics.

**Discussion:**

Birth cohorts are especially suited to elucidate the impact of genetic predisposition, epigenetics, exposures during the first years of life, and gene-environment interactions on the occurrence and progression of asthma and allergies. By building upon an existing cohort, ACROSSOLAR offers a unique and cost-effective opportunity to investigate the aetiology of atopic disease in a prospective and cross-generational way.

## Background

Atopic diseases such as asthma, allergic rhinitis, and atopic dermatitis are the most frequent illnesses among children and adolescents with about one in four children being affected [1, 2]. Their prevalence varies considerably across different regions and populations [3–5]. While asthma and allergies have traditionally been regarded as morbidities of high-income countries, an increase in their prevalence has recently been observed in low- and middle-income countries – a fact that might lead to severe public health problems for the affected regions [2, 6–8]. Moreover, these diseases are also causing substantial economic costs, both in terms of direct costs such as treatments costs and indirect costs such as reduction of work productivity [9, 10].

Concerning the aetiology of these diseases, the current state of knowledge assumes that they are caused by a complex interaction between genetic and environmental factors such as pre- and postnatal maternal smoking or exposure to damp and mould [11–16]. These interactions are understood to be especially powerful during the prenatal period and the first years of life [17–23]. Therefore, these early years are a crucial period when investigating potential risk factors for asthma and allergies. Such investigations should be especially done under consideration of epigenetic mechanisms as these constitute a novel and promising approach to further disentangle the origin and progression of asthma and allergies [24–27]. Out of several potential epigenetic markers, DNA methylation is the most prominent and most frequently investigated one [24, 26–28]. Regarding asthma, there is notable evidence for an association of DNA methylation with various asthma-related phenotypes [29–31]. However, the role of DNA methylation during asthma development as well as underlying biological mechanisms requires further elucidation [26, 32]. Birth cohort studies following the role of DNA methylation prospectively and across generations are the ideal approach to answer these questions [26].

The cross-generational aspect is especially intriguing since there is some first indication of an effect not only of maternal exposures but also of grandmaternal exposures, especially smoking, on subsequent diseases in the offspring [33–36]. Such effects across three generations are also assumed to be mediated by epigenetic modifications [37–39]. Further investigating these aspects can substantially increase the understanding of the aetiology, treatment, and prevention of asthma and allergies [37].

The ACROSSOLAR (Atopic Childhood Disease Risk Factors in the Second Generation of the SOLAR Participants) project described here thus aims at establishing a prospective population-based birth cohort that collects information across three generations. To achieve this aim, the study builds upon an existing cohort which has been recruited for the German branch of Phase II of the International Study of Asthma and Allergies in Childhood (ISAAC) and in the meantime has been followed up twice by the Study on Occupational Allergy Risks (SOLAR) [40–45]. This approach provides the unique opportunity to assess the effects of genetic predisposition, epigenetic factors, and environmental factors such as exposure to environmental tobacco smoke, living conditions, and parental occupation in a prospective and cross-generational study. Accordingly, the main objectives of ACROSSOLAR are:

- To investigate the incidence of childhood asthma and allergies across three generations
- To analyse the association between early-life exposure to potential risk factors such as maternal smoking, maternal stress, or grandmaternal smoking and the incidence of childhood asthma and allergies
- To analyse the impact of epigenetic mechanisms such as DNA methylation on the occurrence of asthma and allergies across generations

The present paper describes the design of the ACROSSOLAR project giving concise and detailed information on the study population, recruitment methods, and study instruments. Furthermore, the challenges in the study design are discussed.

## Methods/Design

### Study design

ACROSSOLAR is a prospective birth cohort study including the members of an already existing cohort and their children.

### Study population

The cohort was recruited originally for the German branch of Phase II of the International Study of Asthma and Allergies in Childhood (ISAAC) [40, 45]. Between 1995 and 1996, a random sample of 7489 children aged nine to eleven years was drawn from the population registries in the cities of Dresden and Munich. Parents of 6399 children (85.3 %) completed a questionnaire on demographic, health-related, and environmental factors. Main outcomes of interest were wheezing, atopic rhinitis, and eczema while the potential risk factors included birthweight, gestational age, breast feeding, family size, family history of diseases, vaccinations, environmental tobacco smoke, physical activity, nutrition, and many more [45]. A sub-sample of the participating children underwent diagnostic tests for asthma symptoms and atopic sensitisation such as skin prick tests (*N* = 4578), skin examination (*N* = 4941), bronchial challenges with hypertonic saline (*N* = 2188), lung function test (*N* = 2589), and measurements of specific and total serum immunoglobulin E (IgE) levels (*N* = 3823) [41, 45, 46].

Those families who had agreed to be contacted again were invited to take part in the first follow-up, the Study on Occupational Allergy Risks (SOLAR), between 2002 and 2003. In total, 3929 adolescents aged 16 to 18 years were included. Using questions from ISAAC Phase II and the European Community Respiratory Health Survey [47], the study examined the course of asthma and atopy and their association with environmental as well as occupational factors such as current state of employment, jobs held during holidays, and the type of job subjects would like to have in the future (preferred job choice) [41, 48]. The jobs indicated by the study subjects were coded according to the International Standard Classification of Occupations-88 [49] and an asthma-specific job exposure matrix was used to classify them as nonexposed jobs, low-risk jobs, and high-risk jobs depending on the level of exposure to potential asthma risk factors [50].

When they were aged 19 to 24 years, 2051 participants took part in the second follow-up, SOLAR II (2007–2009) [42]. Fifty-seven percent of them not only answered the study questionnaire but also underwent clinical examination (including assessment of anthropometric data, blood pressure measurement, skin prick test, patch test, and lung function test). Compared to the previous survey, SOLAR II collected additional information on family status, parenthood, work-related stress, hand eczema, and exposure to cleaning sprays, disinfectants, fumes, and gas. SOLAR II mainly aimed at analysing various potential predictors of work-related sensitisation, allergic rhinitis, and asthma in early work life [43, 44].

**Fig. 1:**
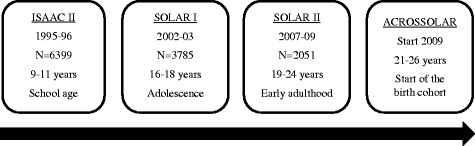
Flow chart of follow-up from ISAAC Phase II to ACROSSOLAR indicating time periods, number of participants, participants’ age, and phase of life

The 2051 SOLAR II participants of whom 58 % are female constitute the eligible population for participating in the ACROSSOLAR study. Figure 1 provides an overview of the course of follow-up from ISAAC Phase II to ACROSSOLAR.

### Recruitment process

Starting in 2009, SOLAR II participants are asked in a bi-annual manner via e-mail if they had conceived a child and, if this is the case, if they are willing to take part in the ACROSSOLAR study. Upon agreement, they then receive an ACROSSOLAR package by mail containing an invitation letter, an informed consent form, and the study questionnaire. To maximise response, we also provide the opportunity to complete the questionnaire electronically via SurveyMonkey (SurveyMonkey Inc., Portland, USA). Therefore, a link to the online version of the questionnaire is sent to the participants in addition to the paper version. As another means of enhancing participation, two postal reminders are sent to non-responders. If participants report parenthood but indicate that they do not want to join ACROSSOLAR, they are not contacted any further.

SOLAR II participants for whom we do not have a valid email address receive a postal invitation together with the informed consent form and the questionnaire. Those participants who changed their residence in the meantime are tracked via the population registry.

**Fig. 2:**
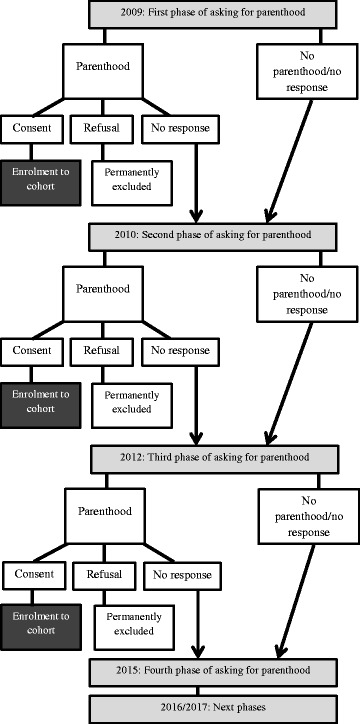
Process of recruitment of those participants with a valid email address

The first invitations were sent out in October and November 2009. From SOLAR II it was known that 44 participants already had a child – the ACROSSOLAR package including the postal and the electronic questionnaire was thus sent out to them directly. 1444 people for whom an email address was available from SOLAR II received an email asking if they conceived a child in the meantime. Those who reported parenthood and agreed to take part in ACROSSOLAR were then enrolled and received the paper and electronic version of the questionnaire. This procedure was repeated in November 2010 when another email was sent to those who had not reported parenthood or had not responded in 2009 (*N* = 1231). The latest period of recruitment was in 2012, when those who had not responded or did not have children so far received another invitation to report parenthood and to enrol in the ACROSSOLAR cohort (Fig. 2). The remaining 563 SOLAR II participants with no valid e-mail address available received the postal package including the invitation letter, the informed consent form and the questionnaire during the above mentioned recruitment phases in 2010 and 2012. The next phase of contacting is planned to be conducted in 2015.

### Sample size

By January 2015, 108 parents completed the paper or electronic version of the questionnaire (73 from Dresden and 35 from Munich). However, the participants were still relatively young when the recruitment for ACROSSOLAR started (aged 21 to 26 years) while in Germany the mean age for giving birth to the first child is estimated to be 29 years [51]. Accordingly, 60 % of the new-borns are conceived by women in the age group between 26 and 35 years [52]. As the majority of the 2051 SOLAR participants will enter that age group during the study period we are expecting a number of about 1000 new-borns being eligible for participation. Based on the experience with SOLAR I and II, we are confident that we can achieve a response rate of 80 %. Hence, we expect the number of returned questionnaires to increase to a total of *N* = 800. For the power calculations, we estimate the prevalence for the main outcome wheezing during the first year of life to lie between 15 and 30 % [8, 53–55], while for the exposure variables (e.g. maternal smoking, maternal stress, grandmaternal smoking) we are reckoning with a prevalence of about 10 %. With 80 % power our sample will then allow us to detect relative risks between 1.57 and 1.91 for 30 and 15 % outcome prevalence respectively.

## Study instruments

### Study questionnaire

The questionnaire involves sections asking for information regarding the child as well as its parents and grandparents. Regarding the child, the questionnaire captures the following information: birth of the child (date of birth, birth weight, type of delivery), medical history in the first year of life and during follow-up in the subsequent years of life (asthma symptoms, diagnosis of airway diseases or atopic dermatitis, intake of medication), breastfeeding (duration of breastfeeding, type of milk used as supplements), and living conditions (number of persons in the household, whereabouts of the child during the day). In each family, the questionnaire is only completed for one child. Those participants who already have more than one child are asked to complete the questionnaire for the youngest one.

The maternal section of the questionnaire asks for the mother’s general health (asthma diagnosis and asthma symptoms, allergies, atopic dermatitis), medical history during pregnancy (infections, gestational diabetes, intake of medication), and smoking. The paternal section is addressed to those fathers who had not participated in SOLAR II (i.e., the mother of the child is a member of the original cohort) and collects information on symptoms or diagnosis of asthma, bronchitis, hay fever, or atopic dermatitis, as well as intake of medication and smoking.

In addition, demographic data (age, education, occupational status, number of children) are recorded for that parent who did not take part in SOLAR II. Moreover, life-time prevalence of asthma, bronchitis, hay fever, and atopic dermatitis among the grandparents of the children is assessed by the questionnaire.

When the children are in primary school age (six to nine years of age), the parents will be asked to complete the questionnaire again. By then, it will be possible to perform a first reliable diagnosis of asthma and atopic dermatitis.

All sections are based on validated questionnaires that have been successfully used in previous studies such as the German Multicentre Allergy Study [56] and the PASTURE project (Protection against Allergy - STUdy in Rural Environments) [57].

### Clinical examination

In addition to the questionnaire data, we aim to invite the children and their parents to a clinical examination when the children are in primary school age. This will involve lung function test and blood sampling. Lung function measurements will be conducted in accordance with the guidelines of the American Thoracic Society [58]. Blood samples will be used for epigenetic analyses and for measurement of total immunoglobulin E (IgE) levels as a test for allergic sensitisation. For analysis of IgE levels, we will use the same procedure as in SOLAR II [42].

Regarding epigenetics, peripheral CD-4+ T cells will be isolated using magnetic cell sorting and isolation kits. DNA will be stored frozen at −80 °C and will be isolated with the FlexiGene DNA Kit (Qiagen NV, Venlo, Netherlands). For array-based DNA methylation analysis, 800 ng of genomic DNA will be treated with sodium bisulfite using the EZ-96 DNA Methylation Kit (Zymo Research Corporation, Irvine, USA). The Infinium HumanMethylation450 BeadChip Kit (Illumina Inc., San Diego, USA) will then be used for measurement of genome-wide DNA methylation. After genome-wide amplification of 4 μl of bisulfite-treated DNA and enzymatic fragmentation, the samples will be added to the chip. The fraction or percent methylation will be reported as Beta-value. This value is expressed as a continuous variable between 0 and 1 corresponding to the ratio of methylated molecules to the sum of methylated and unmethylated molecules [59].

### Data management

Data will be stored and handled at the Institute and Outpatient Clinic for Occupational, Social and Environmental Medicine, University Hospital of Munich (LMU), Munich, Germany. Data from the paper version of the questionnaire will be entered into a password-protected MS Access database (Microsoft Corporation, Redmond, USA). Double data entry will be used to detect and reduce errors. For further analysis, these data will be combined with the questionnaire data that participants directly entered into the SurveyMonkey database. To make full use of all the data collected since 1995, the ACROSSOLAR dataset will also be merged with the datasets of the previous studies (ISAAC II and SOLAR I and II) using a unique study ID.

### Statistical analysis

First, descriptive statistics will outline the characteristics of the study sample. Bivariate analyses will then evaluate potential associations between the exposure and outcome variables that are included in the study questionnaire. Next, statistical methods for analysis of longitudinal data such as Poisson regression and logistic regression models will be used for further examination of possible associations [60, 61]. Potential confounders will be included in the model if they change the effect estimates for the association between the exposure and outcome variables by at least ten percent (change-in-estimate criterion) [62]. To address the problem of missing data, we will run multiple imputations using the R-package Amelia II (The R Foundation for Statistical Computing, Vienna, Austria) which performs expectation-maximization with bootstrapping algorithms [63–65].

Regarding epigenetic analyses, CpG sites that are within 50 bp to known SNPs or in allosomal positions will be removed. For each chip, colour bias adjustment using quantile normalisation and background correction based on a negative control will be performed using the R-package lumi [66]. Further analyses will be carried out with a random intercepts mixed model approach.

### Ethics

The questionnaire part of the study was approved by the Ethical Committee of the Medical Faculty of the University of Dresden, Dresden, Germany. An amendment regarding the epigenetic sampling will be requested in due time.

## Discussion

Atopic diseases not only continue to have a high prevalence in high-income countries, but they are also gaining importance in low- and middle-income countries. They are thus a substantial burden of disease on a global scale. At the same time, it is still not fully understood so far which factors contribute to their development. What has been shown rather reliably is that genetic predisposition, exposures during the first years of life, epigenetic mechanisms, and gene-environment interactions may be crucial. Research aiming at precisely disentangling their role is thus urgently needed and requires innovative methods. Birth cohorts are an approach that is especially suited to tackle this issue [67]. They allow investigating the effect of genetic and environmental factors as well as their interactions on the course of atopic diseases from the early life years onwards.

The ACROSSOLAR study presented here provides the unique opportunity to establish such a birth cohort in the offspring of an existing cohort. Including the parent generation, ACROSSOLAR can also benefit of a wide range of detailed socio-demographic, occupational, and health-related data on the parents and even grandparents of the children that have already been collected during several follow-ups. It is thus possible to follow the course of atopic diseases and potential risk factors across three generations. Another strength of our approach is that not only do we build upon the experience and infrastructure of ISAAC phase II and SOLAR phases I and II but also that the ACROSSOLAR questionnaire is based on validated instruments that have been successfully used in similar projects [56, 57]. Providing a web-based version of the questionnaire is another asset of our study since it has been shown that it becomes increasingly popular among participants to complete surveys online [68]. Also the SurveyMonkey software itself was successfully utilised in previous medical research projects [69–71]. Moreover, integrating epigenetic analysis, namely DNA methylation of isolated CD-4+ T cells, across generations will offer the unparalleled opportunity to investigate underlying mechanisms of asthma development in an unique way.

Regarding potential limitations of our approach, there is some indication from SOLAR II that subjects with higher educational level, higher levels of health consciousness, and higher likelihood of atopic diseases were more likely to participate [42]. This means that the original cohort on which the ACROSSOLAR birth cohort is based might be affected by some form of selection towards higher educated people with higher risk of suffering from asthma or allergies. It seems plausible that the same effect might apply to participation in ACROSSOLAR. On the other side, this could be counterbalanced by the effect that people with lower educational levels conceive children earlier than higher educated people so that more families with lower educational status will enrol into the birth cohort. In any case, the potential of selection bias will be carefully taken into account when analysing and interpreting the collected data. Having said that, it is much more important for the validity of the results in prospective cohort studies to maintain a high level of internal validity [72]. In this context, a possibly more severe form of bias could be introduced by loss to follow-up, especially if it differs between exposed and unexposed subjects [73]. Therefore, maximal effort will be spent to keep loss to follow-up as low as possible, e.g. by sending out two reminders to non-respondents at each phase of follow-up. The same applies to the issue of generalisability: One might argue that especially from a geographic or socio-economic point of view our sample is a rather homogenous group that can hardly be compared with populations in low- and middle-income settings. We are albeit confident that our findings will help to advance the understanding of the underlying biologic principles so that our sample would nevertheless be “biologically representative” [62]. In the past, many important and valid conclusions have been drawn from cohorts that were not geographically representative whatsoever [72, 74].

Another challenge of our study will be to accurately coordinate the enrolment and follow-up of participants as the two will run in parallel. This complex approach requires significant administrative and organisational efforts throughout the study. However, as already mentioned we are building upon the experience and equipment from ISAAC Phase II and SOLAR as well as other large-scale research projects in the field of asthma and allergies.

To conclude, the ACROSSOLAR project offers the unique opportunity to build a birth cohort among the children of an existing large-scale cohort. It allows following the course of asthma, allergic diseases, and a wide range of potential risk factors and underlying mechanisms including epigenetics in a prospective and cross-generational way. The study can thus substantially contribute to further enhance the understanding about the determinants of these diseases.
